# PRL1 and PRL3 promote macropinocytosis via its lipid phosphatase activity

**DOI:** 10.7150/thno.93127

**Published:** 2024-05-27

**Authors:** Zu Ye, Chee Ping Ng, Haidong Liu, Qimei Bao, Shengfeng Xu, Dan Zu, Yanhua He, Yixing Huang, Abdul Qader Omer Al-Aidaroos, Ke Guo, Jie Li, Lai Ping Yaw, Qiancheng Xiong, Min Thura, Weihui Zheng, Fenghui Guan, Xiangdong Cheng, Yin Shi, Qi Zeng

**Affiliations:** 1 Zhejiang Cancer Hospital, Hangzhou Institute of Medicine (HIM), Chinese Academy of Sciences, Hangzhou, Zhejiang 310022, China.; 2 Key Laboratory of Prevention, Diagnosis and Therapy of Upper Gastrointestinal Cancer of Zhejiang Province, Hangzhou, 310022, China.; 3 Institute of Molecular and Cell Biology, A*STAR (Agency for Science, Technology and Research), Republic of Singapore, Singapore 138673.; 4 Human Genome Sequencing Center, Baylor College of Medicine, Houston, TX, 77030, USA.; 5 National Clinical Research Center for Children's Health, Department of Pulmonology of Children's Hospital, Department of Biochemistry, Zhejiang University School of Medicine, Hangzhou 310058, China.

**Keywords:** PRL1, PRL3, lipid phosphatase, macropinocytosis, cancer development

## Abstract

PRL1 and PRL3, members of the protein tyrosine phosphatase family, have been associated with cancer metastasis and poor prognosis. Despite extensive research on their protein phosphatase activity, their potential role as lipid phosphatases remains elusive.

**Methods:** We conducted comprehensive investigations to elucidate the lipid phosphatase activity of PRL1 and PRL3 using a combination of cellular assays, biochemical analyses, and protein interactome profiling. Functional studies were performed to delineate the impact of PRL1/3 on macropinocytosis and its implications in cancer biology.

**Results:** Our study has identified PRL1 and PRL3 as lipid phosphatases that interact with phosphoinositide (PIP) lipids, converting PI(3,4)P_2_ and PI(3,5)P_2_ into PI(3)P on the cellular membranes. These enzymatic activities of PRLs promote the formation of membrane ruffles, membrane blebbing and subsequent macropinocytosis, facilitating nutrient extraction, cell migration, and invasion, thereby contributing to tumor development. These enzymatic activities of PRLs promote the formation of membrane ruffles, membrane blebbing and subsequent macropinocytosis. Additionally, we found a correlation between PRL1/3 expression and glioma development, suggesting their involvement in glioma progression.

**Conclusions:** Combining with the knowledge that PRLs have been identified to be involved in mTOR, EGFR and autophagy, here we concluded the physiological role of PRL1/3 in orchestrating the nutrient sensing, absorbing and recycling via regulating macropinocytosis through its lipid phosphatase activity. This mechanism could be exploited by tumor cells facing a nutrient-depleted microenvironment, highlighting the potential therapeutic significance of targeting PRL1/3-mediated macropinocytosis in cancer treatment.

## Introduction

Phosphatase of Regenerating Liver-3 (PRL3), together with PRL1 and PRL2, form a unique subfamily of protein tyrosine phosphatase (PTP) that is anchored to the cytoplasmic side of endosome and plasma membrane via C-terminal prenylation using C-terminal prenylation motif (CAAX) 1, 2. Overexpression of PRL3 has been linked to cancer metastasis and poor prognosis in various cancer types 2, 3. It has been associated with the activation of key cancer-related signaling pathways, including EGFR, PI3K-AKT, and mTOR, as well as the downregulation of PTEN and the promotion of epithelial-mesenchymal transition (EMT) 2, 4, 5. Furthermore, PRL3 has been found to be involved in autophagy-mediated ovarian cancer progression 6. PRL3 shares limited sequence homology with the lipid phosphatase PTEN, and indeed, PRL3 has been shown to function as a lipid phosphatase, dephosphorylating phosphoinositides (PIPs), including PI(4,5)P_2_ and PI(3,4,5)P_3_ in vitro 7. This discovery places PRL3 among a growing list of PIP-binding proteins that lack the standard binding domains 8, 9. Nevertheless, the precise biological ramifications of PRL3 acting as a lipid phosphatase remain largely unresolved.

Cellular homeostasis is paramount for normal development. Cellular processes involved in maintaining homeostasis encompass the regulation of factors such as temperature, intercellular communication, and the synthesis and degradation of molecules. Disruptions in these mechanisms can lead to a range of diseases, including disorders in the nervous system, cardiac system, and metabolic system like diabetes, as well as cancer. Remarkable progress has been made in understanding the protein machinery governing these processes. However, it is only in the last two decades that the role of lipids in orchestrating diverse cellular events has gained recognition 10, 11. This newfound perspective has also uncovered links between lipid metabolism and a variety of human diseases. Notably, one crucial class of lipids in this context is the family of PIPs 11, 12.

PIPs are lipid second messengers derived from phosphatidylinositol (PI) via phosphorylation at the inositol ring's hydroxyl groups at positions 3, 4, and/or 5, generating seven distinct PIPs, contributing to their signaling diversity 11, 12. They play pivotal roles in regulating many cellular processes such as protein trafficking, cytoskeletal rearrangement, migrasome formation, cell growth, proliferation, and motility 12-15. Aberrant PIP signaling has been implicated in a multitude of human diseases, including cancer, neurological disorders, channelopathies, and diabetes 11-14, 16. PIPs also act as signaling molecules themselves, recruiting protein regulators to specific cellular sites and contributing to organelle identity and functionality. They fulfil their functional roles by associating with numerous coordinators, such as adaptors, protein kinases, and small guanosine triphosphatases (GTPases) that contain specialized PIP binding domains such as PH domain, PX domain, and FYVE domains 17-20.

Different species of PIPs are dynamically interconverted by the coordinated action of many kinases and phosphatases 11, 12. These enzymes are tightly coupled to maintain appropriate levels of PIPs and any deregulation in their activity leads to many diseases including cancer. For example, PTEN is a lipid phosphatase converting PI(3,4,5)P_3_ into PI(4,5)P_2_ to antagonize PI3-kinases 7, 21, 22. PTEN is among the most mutated tumor suppressors, and its mutations generally inactivate its lipid phosphatase activity, regulating increasing levels of PI(3,4,5)P_3_ for sustainable signaling (such as the AKT pathway), similar to activating mutations of PI3-kinases 21-23.

Macropinocytosis is a nutrient procurement pathway that aids cells in extracting nutrients from the extracellular environment, fuelling cell growth 24-26. The initial and critical step in macropinocytosis involves membrane ruffling or blebbing, which appears to be regulated by various PIPs 25, 27. Inhibitors of phosphoinositide 3-kinases (PI3Ks), responsible for generating PI(3,4,5)P_3_ from PI(4,5)P_2_, can impair macropinosome formation 28. Additionally, knockdown of inositol polyphosphate 4-phosphatase type II (INPP4B), which specifically dephosphorylates PI(3,4)P_2_ to generate PI(3)P, can suppress the macropinocytic uptake of extracellular solutes 29. The dynamic changes in PIPs during macropinocytosis have been described in various cell types. In EGF-stimulated A431 cells, the PI(4,5)P_2_ level increases in membrane ruffles, reaching its maximum before circular ruffle formation, and rapidly falling afterward 30. In contrast, the PI(3,4,5)P_3_ level increases in circular ruffle formation and peaks at the beginning of circular ruffle fusion 31, 32. In colony-stimulating factor-stimulated macrophages, transient and sequential spikes of PI(4,5)P_2_, PI(3,4,5)P_3_, PI(3,4)P_2_, and PI(3)P in membrane ruffles are observed during macropinocytosis 33. It is increasingly being recognized that increased macropinocytosis is being utilized by cancer cells for their growth in nutrient limiting microenvironment 24.

Our current study has uncovered a novel function of PRL1 and PRL3 as lipid phosphatases, generating PI(3)P from PI(3,4)P_2_ and PI(3,5)P_2_. This function is closely associated with their ability to promote membrane blebbing and macropinocytosis. Since PRL1 and PRL3 are overexpressed in diverse cancer cells and cancer types 34, the PRLs-induced macropinocytosis may contribute to enhanced nutrient intake, supporting the accelerated metabolism observed in cancer cells. Our findings indicate that PRL1/3 orchestrates nutrient sensing, absorption, and recycling within cells, which is particularly advantageous for tumor cells residing in nutrient-depleted microenvironments. As a result, targeting PRL3 presents an important therapeutic strategy in the context of cancer treatment, as evidenced by recent clinical trials using PRL3-zumab, the first-in-class humanized antibody targeting intracellular PRL3 35, 36. PRL3-zumab has shown its excellent drug safety since its 2017 Phase 1 Clinical Trial at the National University Hospital Singapore (NUHS). PRL3-zumab Phase 2 Clinical trials have been approved by multiple national authorities, including the HSA (Singapore), FDA (US), NMPA (China), and NPRA (Malaysia). An online article reported that PRL3-zumab extended the life expectancies of a late-Stage IV Gastric cancer patient by years, showing PRL3-zumab could stand behind PD1-PDL1 non-responders as a rescue immunotherapy 37. PRL3 and PRL3-zumab represent a coming new era of cancer immunotherapies.

## Results

### Overexpression of PRL1 or PRL3 disrupts of the cellular membrane

**Figure 1:**
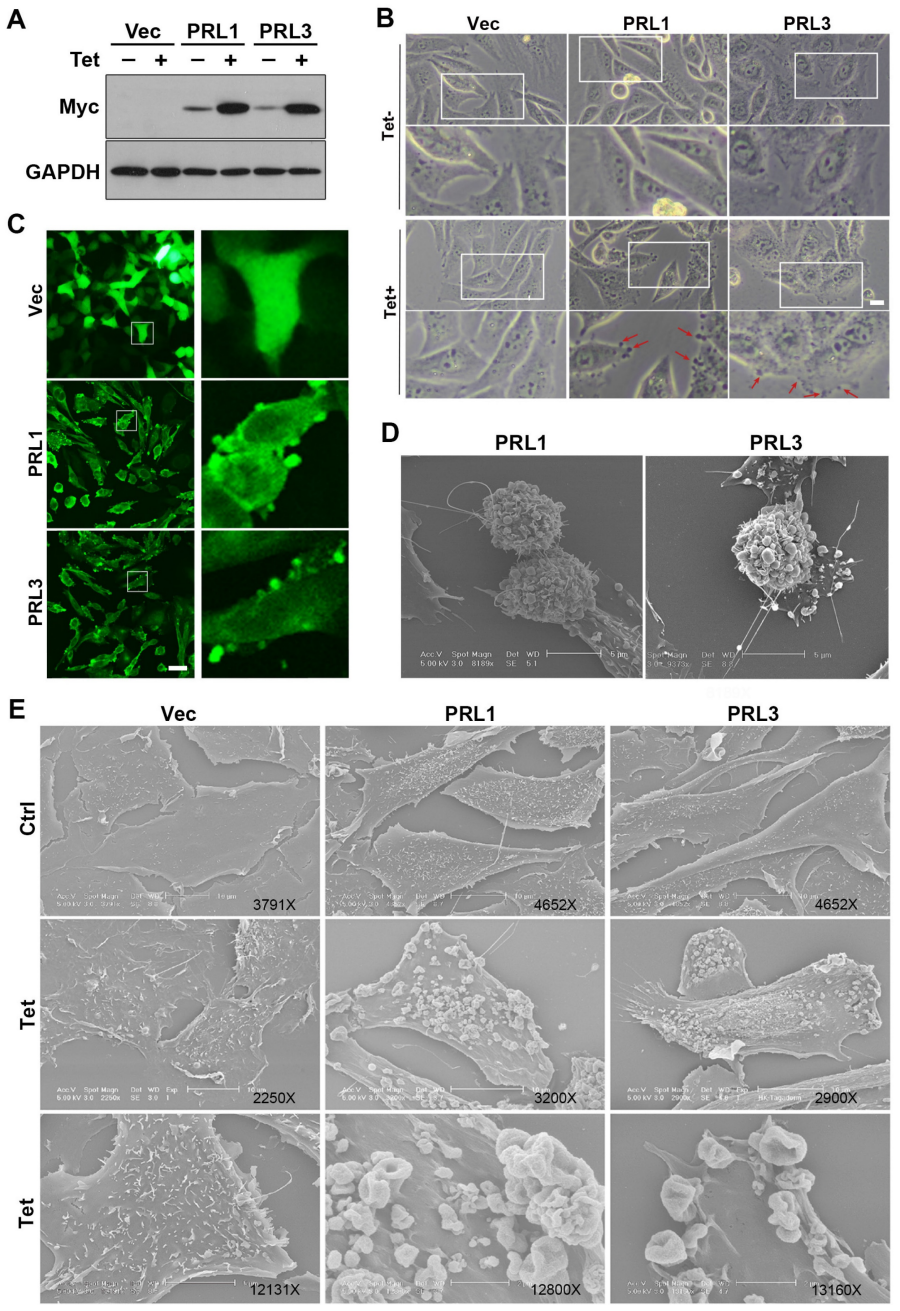
** Overexpression of PRL1 and PRL3 induces membrane blebbing.** (**A**) CHO cells for over-expression of myc-PRL1 and myc-PRL3 were treated with or without 4 µg/ml of doxycycline-HCl for 4h (labeled as Tet). Cell lysate was then subjected to western blot analysis with myc antibodies. (**B**) Cells as described in panel A were observed under the light microscope. Scale bar, 10 µm. (**C**) Cells as described in panel A with the Tet treatment were immunostained with myc (green). Scale bar, 50 µm. (**D-E**) Cells described as in panel A were observed under electron microscopy (EM). Scale bars were as labeled.

The lipid bilayer has fluidity and self-sealing properties. This incredible behavior, fundamental to the creation of a living cell, follows directly from the shape and amphipathic nature of the phospholipid molecule. We previously established stable Chinese hamster ovary (CHO) cell lines over-expressing myc-PRL1 or myc-PRL3 under the control of a tetracycline-inducible promoter 38. Treatment of cells with tetracycline induced significant upregulation of PRLs compared to untreated controls (**Figure 1A**). Interestingly, our investigation revealed a striking phenomenon: induced overexpression of PRL1 or PRL3 resulted in the appearance of significant membrane blebbing in CHO cells, a phenomenon not observed in control cells (**Figure 1B**). Then we used immunostaining of myc on these stable cells to observe the distribution of PRL1 or PRL3. We found that the myc-PRL1 or myc-PRL3 signal decorated the plasma membrane with punctate structures, consistent with the membrane blebbing morphology observed (**Figure 1C**). Intriguingly, scanning electron microscopy (SEM) further unveiled the extent of membrane disruption caused by PRL1 or PRL3 overexpression. SEM images (**Figure 1D-E**) revealed significant membrane ruffling and cup-like structures, reminiscent of macropinocytic or phagocytic cups. The presence of numerous mushroom-like structures of varying sizes was notable, suggesting a drastic or aggressive alteration in membrane morphology associated with macropinocytosis.

These observations shed light on the impact of PRL1 or PRL3 overexpression on the cellular membrane. The aggressive changes in membrane morphology, particularly the formation of membrane blebs and protrusions, provide intriguing insights into the potential roles of these phosphatases in membrane dynamics and cellular function.

### PRLs interact with PIPs

**Figure 2:**
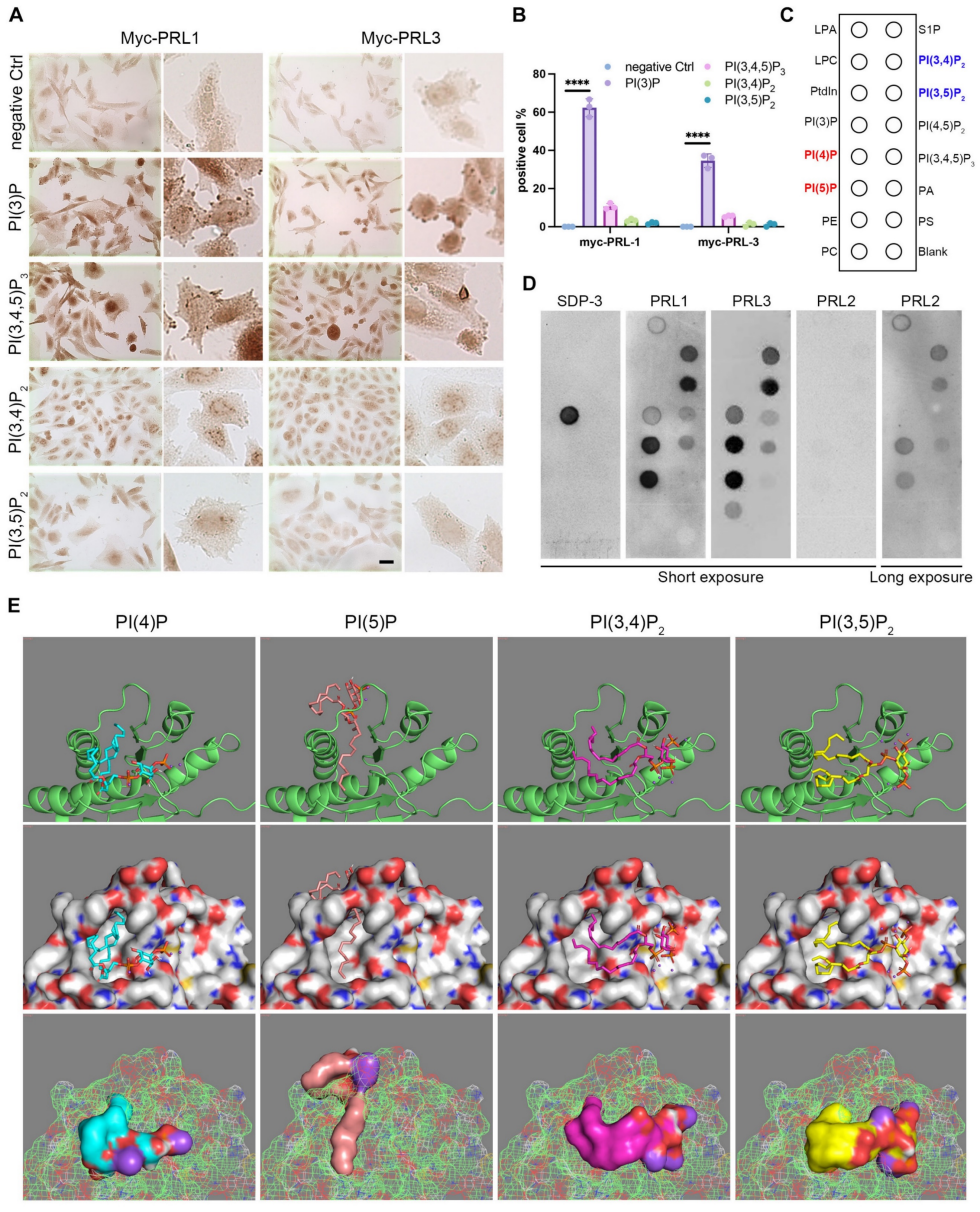
** PRLs interact with PIPs.** (**A-B**) Immunohistochemical (IHC) staining for different PIP lipids in CHO cells with over-expression of myc-PRL1 and myc-PRL3. Scale bar, 50 µm (**A**). Statistical analyses were detailed in panel (B), where the percentage of cells exhibiting positive immunohistochemical (IHC) staining was quantified (mean ± SD). ****p < 0.0001. (**C-D**) PIP lipid strip for interaction with purified GST-PRL1, GST-PRL3 and GST-PRL2. SPD-3 was a positive control with a specific affinity to PI(3)P. (**E**) Molecular docking analysis of PRL3 with 4 interacting PIP lipids detected in panel D.

In light of the active influence of membrane lipids on membrane dynamics, we embarked on an investigation to explore the potential role of membrane lipids in the context of PRL1 and PRL3-induced membrane blebbing and ruffling. The composition of membrane lipids can significantly impact cellular processes, making this investigation of particularly relevant. Through immunohistochemistry, we made a noteworthy discovery. Among the PIPs analyzed, only PI(3)P exhibited a significant enrichment in membrane blebs (**Figure 2A-B**). This observation hints at the pivotal role of PI(3)P influenced by PRLs in the membrane blebbing phenomenon (**Figure 1**). Additionally, our previous findings on PRL1 and PRL3's enrichment in membrane blebs (**Figure 1C**) raised the intriguing possibility of PRL phosphatases' interaction with PIPs, particularly PI(3)P, at the cell membrane.

To delve further into this possibility, we conducted experiments involving GST-PRL1 and GST-PRL3 recombinant proteins incubated on PIP lipid strips (**Figure 2C**). Surprisingly, PI(3)P exhibited low affinity to PRL3 protein. Instead, PI(4)P, PI(5)P, PI(3,4)P_2_ and PI(3,5)P_2_ were found to bind strongly to PRL3 (**Figure 2C-D**). These interactions were further substantiated through bioinformatics docking analysis (**Figure 2E**).

The docking analysis provided computational validation of the interactions between PRL3 and PI(4)P, PI(5)P, PI(3,4)P_2_, and PI(3,5)P_2_, supporting our earlier findings. These results suggest that PRL3 has a high affinity for these PIP lipids, paving the way for a more comprehensive understanding of how PRL3 may play a role in modulating membrane dynamics and cellular processes through these lipid-protein interactions.

### PRL1 and PRL3 act as lipid phosphatases

**Figure 3:**
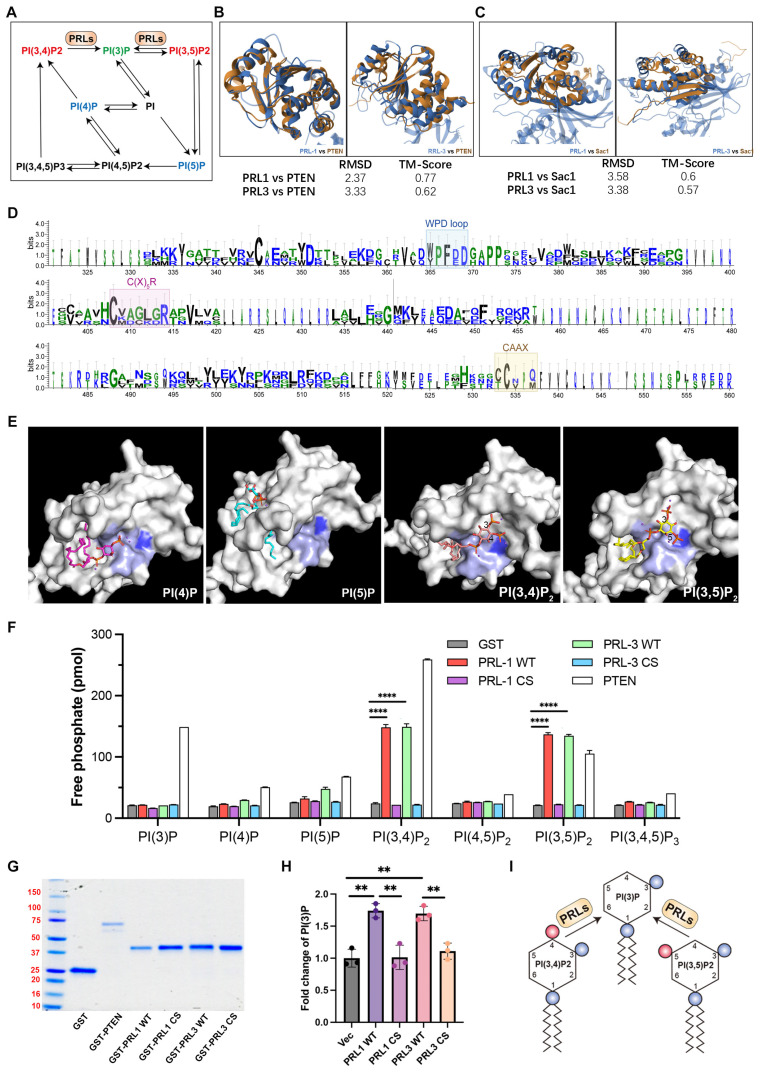
** PRL1 and PRL3 act as lipid phosphatases.** (**A**) A simplified cartoon showing the relationship among different PIP lipids. (**B**) Comparison of the structures of PRL1 (PDB code: 1RXD) or PRL3 (PDB code: 1R6H) and PTEN (PDB code: 1D5R) (**C**) Comparison of the structures of PRL1 or PRL3 and Sac1 (PDB code: 3LWT). (**D**) Alignment of sequences of PRL1, PRL3, PTEN and SAC1 proteins. The size of the single letters in the sequence scales with its conservation at this position. The conserved motif required for PRLs' enzyme activity is highlighted by labeled rectangles. The figure was presented through http://weblogo.threeplusone.com/. (**E**) Molecular docking analysis showing the 4 interacting PIP lipids docking into PRL3. (**F**) Malachite green assay to confirm the lipid phosphatase activity of PRL1/3. Data are from three replicate experiments (mean ± SD). (**G**) Coomassie blue staining of purified proteins as indicated. (**H**) PI(3)P in cells with overexpression of PRL1/3 WT or CS mutant were measured by a quantitative ELISA. The PI(3)P levels were normalized to those observed in cells expressing empty vector. **p< 0.01. (**I**) A cartoon showing the direct dephosphorylation of PI(3,4)P_2_ and PI(3,5)P_2_ by PRLs.

Both PI(3,4)P_2_ and PI(3,5)P_2_ can be dephosphorylated to become PI(3)P (**Figure 3A**). PI(3)P was observed enriching in membrane blebs and there are direct interactions between PRLs and PI(3,4)P_2_ or PI(3,5)P_2_, we therefore proposed that PRL1 and PRL3 may function as lipid phosphatases, converting PI(3,4)P_2_ and PI(3,5)P_2_ to PI(3)P at the sites of membrane blebbing and ruffling. This hypothesis gains support from several lines of evidence.

Firstly, we noted significant similarities between PRL1 and PRL3 and two well-identified lipid phosphatases, PTEN and SAC1. These similarities encompass both sequence and structural features (**Figure 3B-D**). Notably, the protein sequences of PRL1, PRL3, PTEN and SAC1 exhibit conservation within the catalytic loop, including the WPD loop, C(X)_5_R and CAAX (**Figure 3D**).

Our molecular docking analysis further reinforced this hypothesis. We found that PI(3,4)P_2_ and PI(3,5)P_2_ were closely docking into the conserved phosphatase catalytic loop (**Figure 3E**). Specifically, the 3' phosphate groups of both PI(3,4)P_2_ and PI(3,5)P_2_ were situated in close proximity to a key amino acid (C104) for the phosphatase activity of PRLs. In contrast, PI(4)P and PI(5)P did not exhibit similar close interactions with the catalytic loop (**Figure 3E**), increasing the likelihood of lipid phosphatase activity.

To further confirm lipid phosphatase activity of PRL1 and PRL3, we purified the GST-labeled PRL1 or PRL3 (both WT and a known catalytically-inactive mutant C104S) and then utilized the malachite green assay to detect the release of phosphates upon incubation of PIs with PRL1 and PRL3 (**Figure 3F-G**). This assay detects the release of phosphates when PIs are incubated with PRL1 or PRL3. Our data unequivocally demonstrated that the incubation of purified PRL proteins with PI(3,4)P_2_, PI(3,5)P_2_, and PI(3,4,5)P_3_ resulted in a significant increase in phosphate disengagement from the PIs (**Figure 3F-G**). Notably, the catalytically-inactive mutant of PRLs (C104S) abolished phosphatase activity (**Figure 3F-G**). To validate that PRLs catalyze the conversion of PI(3,4)P_2_ and PI(3,5)P_2_ into PI(3)P, rather than PI(4)P or PI(5)P, we conducted a quantitative ELISA assay to measure cellular PI(3)P concentrations. Our results indicate that expression of PRL1/3 wild-type WT significantly increased PI(3)P levels, whereas the vector or PRL1/3 CS mutant did not produce a similar effect (**Figure 3H**). This result confirms that, in addition to their established roles as dual specificity phosphatases, PRL1 and PRL3 indeed function as lipid phosphatases to act on PI(3,4)P_2_ and PI(3,5)P_2_ to generate PI(3)P, thereby leading to a localized increase in PI(3)P concentration within the cellular membrane (**Figure 3I**).

### PRLs interactomes identify extracellular proteins

**Figure 4:**
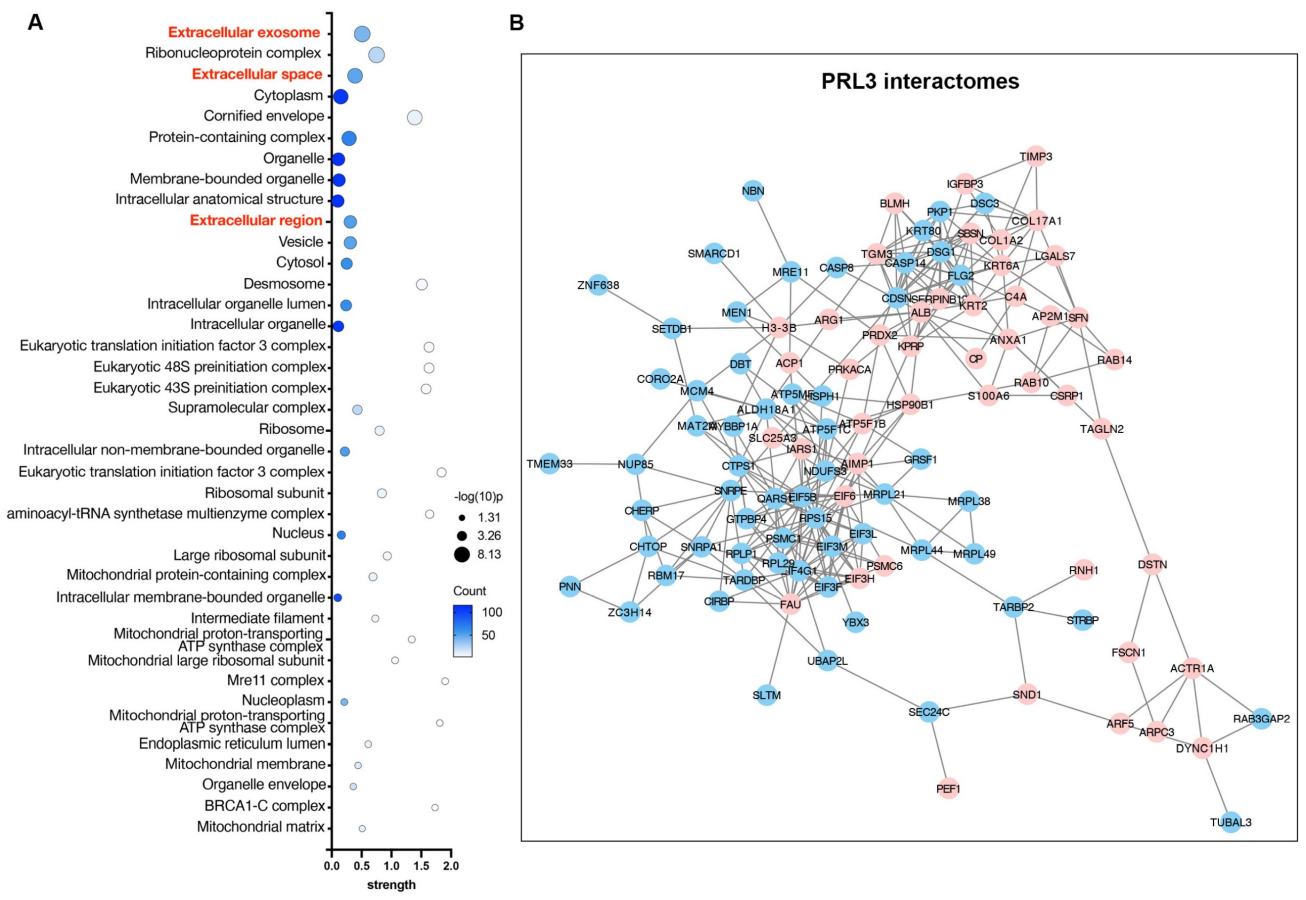
** PRL3 interactomes identify extracellular proteins.** (A) GO analysis (cellular compartment) on the PRL3 interactomes. (B) The interactomes network map of PRL3 interactomes. The red dots show the proteins known to be involved in extracellular region regulation.

To understand the function of PRLs as lipid phosphatases, we identified and analyzed the protein interactomes of PRL3 via co-immunoprecipitation of GFP-PRL3 overexpressed in HeLa cells, followed by mass spectra proteomics analysis. We identified 154 proteins interacting with PRL3. Since we focus on the phosphatase function on the unique membrane structures we identified (**Figure 1**), all interactome proteins were analyzed for their distribution in cellular compartments by performing a gene ontology (GO) analysis. We identified a group of gene ontology terms related to extracellular regulation, including extracellular exosome, extracellular space, and extracellular region (**Figure 4A-B**). The membrane blebbing and circular ruffles were found in the PRLs overexpressed cells. Therefore, the lipid phosphatase activity of PRLs may be associated with macropinocytosis, which is featured with the membrane ruffling and allows cells to extract nutrients from extracellular sources 24, 25, we then analyzed the role of PRL3 on macropinocytosis.

### PRLs induce macropinocytosis depending on the phosphatase activity

**Figure 5:**
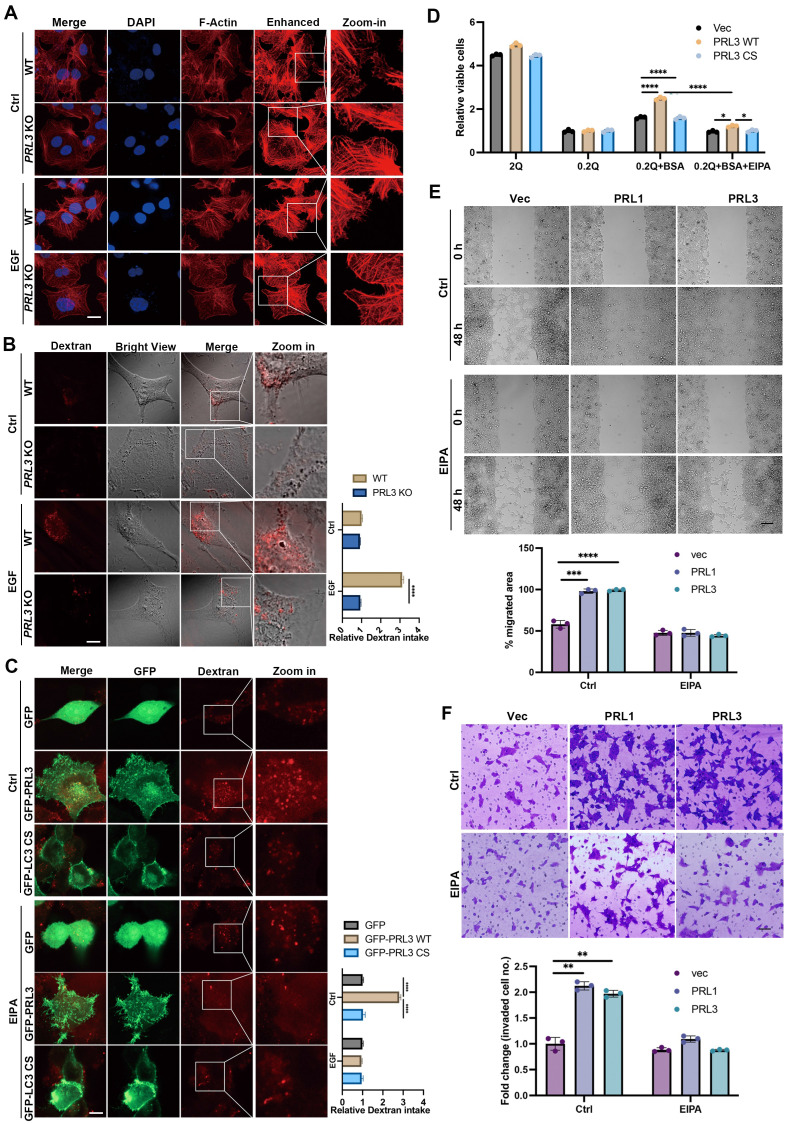
** PRLs induce macropinocytosis depending on the phosphatase activity.** (**A**) WT and PRL3 KO Huh7 cells were treated with or without EGF (200 ng/mL). The IF signal of F-actin was observed under confocal microscopy. Scale bar, 10 μm. The lane labeled "Enhanced" was included to display the digitally enhanced signal of F-actin staining, providing a clearer visualization of membrane morphology. (**B**) Dextran 70KD uptake assay was performed on WT and PRL3 KO U251 cells with or without treatment of EGF (200 ng/mL). Scale bar, 10 μm. (**C**) Dextran 70KD uptake assay was performed on U251 cells overexpressing GFP, GFP-PRL3 and GFP-PRL3 CS mutant. Cells were treated with or without macropinocytosis inhibitor EIPA. Scale bar, 10 μm. (**D**) Proliferation of indicated cells incubated in medium with normal (2 mM Q) or subphysiological (0.2 mM Q) glutamine concentration in the absence or presence of 2% BSA. (**E**) Wound healing assay of the Vec, myc-PRL1 and myc-PRL3 overexpressing cells. Cells were treated with or without EIPA. Scale bar, 50 μm. (**F**) Transwell assay of the Vec, myc-PRL1 and myc-PRL3 overexpressing cells. Cells were treated with or without EIPA. Scale bar, 50 μm.

To confirm the involvement of PRL1/3 in macropinocytosis, a process mediated by actin filaments, we investigated actin dynamics in PRL3 knockout cells. We found the actin-positive protrusions on cellular membranes were significantly decreased in the PRL3-depleted cells, even when a macropinocytosis inducer EGF was used (**Figure 5A**). This observation hints at PRL3's role in actin dynamics during macropinocytosis process. We then co-stained the F-actin with GFP-PRL1/3 to observe their distributions. Co-staining of F-actin with GFP-PRL1/3 provided compelling evidence for the colocalization of GFP-PRL1/3 with F-actin, particularly in regions with membrane blebbing structures (**Figure S1A, upper panel**). Additionally, these specialized membrane blebbing structures, as indicated by double labeling with F-actin and GFP-PRL1/3, were notably absent when cells were treated with an EGFR inhibitor, erlotinib, which is reported to block macropinocytosis (**Figure S1A, lower panel**). These findings strongly support the notion that PRL1/3 is intricately involved in the promotion of macropinocytosis.

To further substantiate this, we quantified macropinocytosis by measuring the cellular uptake of tetramethylrhodamine-labeled high-molecular-mass dextran (Dextran 70KD). We knocked-out PRL3 expression in three different cell lines: liver cancer Huh7 cells and two glioma cell lines U87 and U251 cells (**Figure S1B**). We found that ablation of PRL3 in these cell lines all significantly inhibited the intake of Dextran 70KD in both basal or EGF-induced macropinocytosis conditions (**Figure 5B & S1C-D**), indicating the requirement of PRL3 in basal and EGF-induced macropinocytosis. Consistently, glioma cells (U251 & U87) over-expressing GFP-PRL3 exhibited increased Dextran 70KD uptake, indicative of enhanced macropinocytosis (**Figure 5C & S1E**). These effects were almost entirely negated when cells were treated with the macropinocytosis inhibitor, 5-(N-ethyl-N-isopropyl) amiloride (EIPA) (**Figure 5C & S1E**). A similar trend of heightened Dextran uptake was observed upon overexpression of myc-PRL1 or myc-PRL3 in CHO cells (**Figure S1F**). Furthermore, overexpression of the catalytically inactive mutant PRL3 C104S (PRL3 CS) failed to induce increased Dextran 70 KD uptake (**Figure 5C & S1G**). These observations conclusively demonstrate the role of PRL3 in driving macropinocytosis, a function dependent on its phosphatase activity.

Given that macropinocytosis is recognized for its contribution to cancer development by facilitating the uptake of nutrients to support cell proliferation 24, 39, we investigated how PRL1 and PRL3 might function under conditions of nutrient deprivation. We imposed a glutamine-deficient culture (0.2Q), which substantially inhibited cell growth in all three cell lines (**Figure 5D**). Upon supplementation with an additional 2% bovine serum albumin (BSA), cells overexpressing PRL1/3 wild-type (WT) showed a partial restoration of cell proliferation compared to cells expressing catalytically inactive mutants. Interestingly, this phenomenon was largely abrogated by the macropinocytosis inhibitor EIPA (**Figure 5D**). These results underscore the role of PRL1 and PRL3 in promoting macropinocytosis to exploit exogenous nutrients to sustain cell proliferation under glutamine deprivation.

In addition to its impact on nutrient uptake and cell proliferation, the promotion of macropinocytosis was associated with enhanced cell migration and invasion. This was evident in wound healing and invasion assays performed in PRL1/3-overexpressing cells (**Figure 5E-F**). The PRL1/3 overexpression significantly increased wound healing speed and invaded cell numbers, while macropinocytosis inhibitor EIPA almost totally stopped these phenomena (**Figure 5E-F**). These findings align with the established roles of macropinocytosis in cell migration and invasion 39-42, reinforcing the multifaceted implications of PRL1 and PRL3 in cancer progression.

### PRLs are involved in glioma development

**Figure 6:**
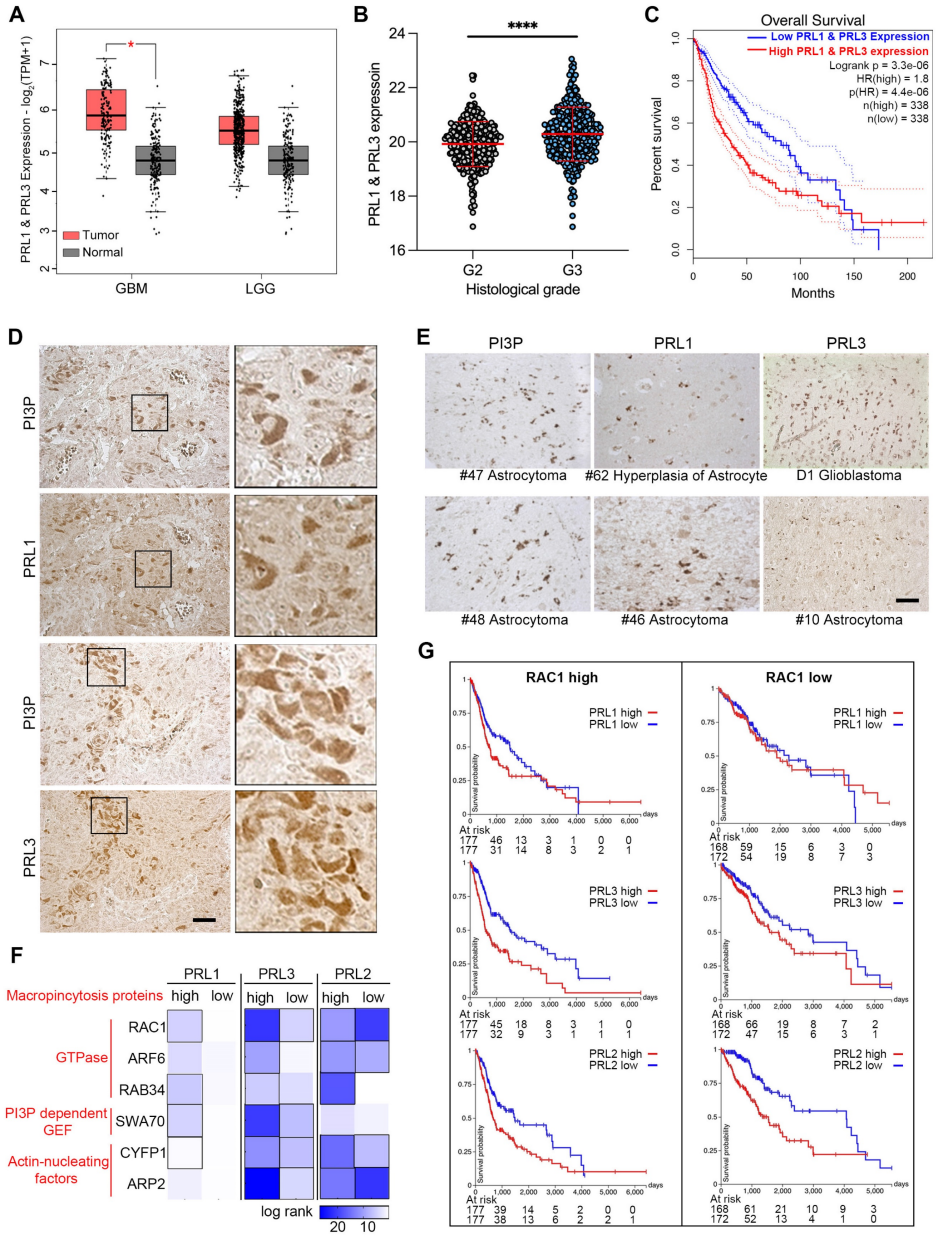
** PRLs are involved in glioma developments.** (**A**) The expression data of PRL1 and PRL3 retrieved from TCGA and GTEx database. Red bar, tumor samples. Grey bar, normal samples. Cancer entities acronyms: Glioblastoma multiform (GBM), Brain Lower Grade Glioma (LGG). (**B**) The PRL1 and PRL3 normalized expression levels (log_2_(Norm_Count+1)) in GBM and LGG samples were compared by their histological Grade. (**C**) Kaplan-Meier survival curves for LGG and GBM samples were presented. (**D-E**) Immuno-histochemical staining of PRL1, PRL3 and PI(3)P on brain tumor tissues. (**F**) Overall survival map for PRL3 and macropinocytosis genes was generated based on the TCGA datasets (GBM & LGG). The dark blue boxes denote higher risk and the white boxes represent lower risk, with an increase in the gene expression. The blocks with black frames indicate statistical significance in prognostic analyses. (**G**) TCGA GBM and LGG samples were divided into two groups with RAC1 high expression or low expression. Kaplan-Meier survival curves for indicated PRL genes were presented.

We also examined the direct interaction of PRLs with PIPs and the novel lipid phosphatase function involved in macropinocytosis, which depends on membrane lipid changes. Since the brain tissue contains 50% of the total body lipids, and PRL3 is reported to be increasingly expressed in higher Grade glioma tissues 43, we are curious to know the function of PRLs in brain cancer development. Initially, we compared the mRNA level of PRL1 and PRL3 in normal and glioma samples sourced from a public database. Our analysis revealed an increasing trend in the expression of PRL1 and PRL3 in tumor samples, although this trend was not statistically significant in low-grade gliomas (LGG) (**Figure 6A**). Furthermore, consistent with previous reports, we observed higher expression levels of PRL1 and PRL3 in histological Grade III samples compared to Grade II samples (**Figure 6B**). Kaplan-Meier survival analysis further showed that patients with higher PRL1 and PRL3 expression had a significantly lower probability of overall survival (OS) (**Figure 6C**). Therefore, our findings suggest that the expression of PRL1 and PRL3 may play a promotive role in glioma development.

We utilized immunohistochemistry to label PRL1, PRL3 and PI(3)P in glioma tissues. The PRL1-, PRL3- or PI(3)P-positive cells were found in almost all of the glioma tissues (**Figure 6D-E**). Interestingly, we observed a strikingly similar distribution pattern between PRLs (PRL1 or PRL3) and PI(3)P in several tumor tissues (**Figure 6D**), suggesting the possibility that the highly expressing PRLs may lead to PI(3)P accumulation in cancer cells.

Macropinocytosis, as we have shown to be up-regulated by the overexpression of PRLs, plays a critical role in assisting cancer cells in meeting the high demands for energy and nutrients within the limited tumor microenvironment 24, 25. To unravel the function of PRLs-mediated macropinocytosis in the context of glioma development, we conducted an expression analysis correlated with cancer patient survival.

We initiated by generating a heatmap of log-rank values for OS based on the expression levels of both PRLs and genes associated with macropinocytosis (**Figure 6F**). This analysis aimed to shed light on the interplay between PRLs and the molecular machinery required for macropinocytosis. The heatmap revealed intriguing trends—patients expressing higher levels of macropinocytosis-related genes appeared to have longer survival times when the expression of PRL1 or PRL3 was lower (**Figure 6F-G**). For instance, PRL1 and PRL3 exhibited more substantial effects on the OS of patients with highly expressed RAC1, a critical gene required for macropinocytosis, whereas the impact was less pronounced in patients with lower RAC1 expression levels. Interestingly, as we previously demonstrated, PRL2 did not exhibit interactions with PIPs (**Figure 2C-D**). In the survival heatmap, we noted that while PRL2 had some noteworthy effects on patients' OS, the survival trends for PRL2 did not consistently align with macropinocytosis-related gene categorizations, unlike PRL1 or PRL3 (**Figure 6F-G**). These findings provided additional evidence suggesting a possible association between PRL1/PRL3 and macropinocytosis concerning their influence on cancer prognosis.

## Discussion

**Figure 7:**
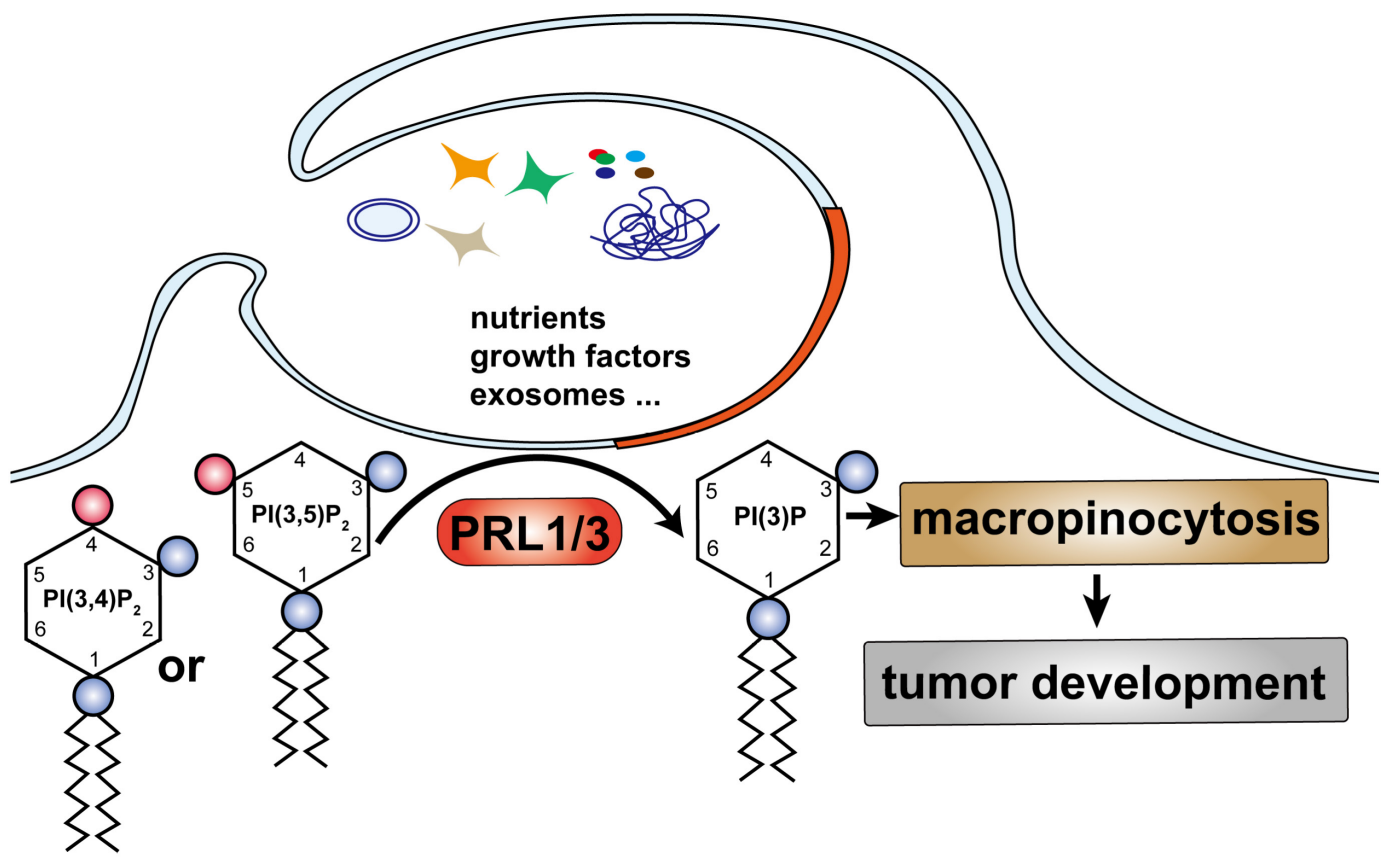
** A simplified model describes our finding:** PRL1/3 generates PI(3)P on the plasma membrane, leading to membrane blebbing and then curving to allows tumor cells to utilize macropinocytosis to support their development.

Building upon our previous research, which highlighted the critical role of type III phosphatidylinositol 3-kinase PIK3C3 in PRL3-mediated autophagosome formation 6, we set out to investigate the involvement of PRL3 in the production of PI(3)P, a lipid with established functions in endosomal fusion, protein sorting, and autophagy 44-47. In this study, we unveil a novel role for PRL1 and PRL3 but not PRL2 as inositol phosphatases with the specific capacity to enrich PI(3)P levels by dephosphorylating PI(3,5)P_2_ and/or PI(3,4)P_2_ substrates (Fig 7). This discovery expands our understanding of PIPs and their integral roles in fundamental cellular processes. One striking observation is the potentiation of macropinocytosis in cells with elevated PRL1/3 expression, presenting an alternative mechanism for nutrient uptake, which, in turn, supports cell survival, fosters migration, and fuels invasion. This finding underscores the vital role of PRL1/3 in cancer development.

Importantly, PI(3)P has recently been recognized by our previous work as a promoter of lamellipodia formation and a key player in activating mTOR signaling 5, 48. Additionally, lysosomal PI(3)P has been implicated in marking the motile mTORC1 signaling, thus regulating cellular adaptation to fluctuating nutrient supplies 49. The current study, revealing PRL3-mediated PI(3)P generation, offers an alternative perspective on the PRL3-mTOR signaling axis and provides further support for the positive influence of PI(3)P on mTOR activation. However, an intriguing question arises concerning the known localization of both PRL3 and PI(3)P on late endosome/lysosome membranes 5, 49, 50. This prompts us to investigate whether PRL3 also functions as a lipid phosphatase on lysosomal membranes to generate PI(3)P in future studies. Additionally, PI(3)P is also considered to be important for autophagy regulation through modulating membrane dynamics to support autophagsome formation, recruiting autophagy machinery proteins, and promoting autophagosome formation 51-53. Combined with our previous findings showing a positive role of PRL3 in autophagy process 6, it should be interesting to systematically explore the role of PRL1/3-induced PI(3)P generation in autophagy regulation in the future.

PIPs such as PI(4,5)P_2_ and PI(3,4,5)P_3_ have been well-recognized for their roles in cancer 54-57. Recently, in PIK3CA-mutant ER+ breast cancer cells, the lipid phosphatase INPP4B was identified as a promoter of cancer development through the generation of PI(3)P on late endosome/lysosome membranes, subsequently inducing Wnt/β-catenin signaling to drive cell proliferation 58. However, the specific function of plasma membrane-accumulated PI(3)P in cancer growth has remained largely unexplored. Our findings hint at a potentially novel and direct role for PI(3)P in cancer by promoting macropinocytosis via enhancing plasma membrane PI3P levels, warranting further investigation.

Moreover, the induction of membrane ruffling or blebbing by PRL1/3-mediated accumulation of PI(3)P on the plasma membrane points to the promotion of macropinocytosis. Recent studies have highlighted the potent role of macropinocytosis in cancer development, with cancer cells utilizing this process to enhance cell migration, invasion, and nutrient acquisition 24, 25, 39-41. Consistent with these findings, our study demonstrates that PRL1/3-induced PI(3)P membrane accumulation enhances cell migration, invasion, and nutrient uptake by promoting macropinocytosis.

Our previous work has also identified PRL3 as a positive regulator of autophagy, a cellular process that plays a crucial role in supporting cancer cells residing in nutrient and energy-deprived microenvironments 6, 59. The current study adds another dimension to the multifaceted roles of PRL3 by revealing its capacity to stimulate macropinocytosis, which, as shown here, can partially rescue cell proliferation under low-glutamine conditions when supplemented with additional proteins, such as BSA. A recent study has identified macropinocytosis as a compensatory mechanism for autophagy-compromised cancer cells to maintain their survival in a microenvironment with limited nutrients supply 24. Therefore, PRL3 maybe important for energy generation in both autophagy-defetient or proficient cancer cells, thereby supporting cancer cell survival in challenging conditions.

Our group has already developed the PRL3-zumab, a first-in-class humanized antibody against PRL3, as a promising approach to anticancer therapy 60, 61. Our study offers essential evidence bolstering the rationale for specific antibody-targeted therapy against PRL3, underlining its potential as a valuable strategy in the fight against cancer. In view of current finding, cancers relying on macropinocytosis may be more beneficial from PRL3-zumab as a novel cancer immunotherapy.

## Material and methods

### Reagents and antibodies

The reagents used in this study were as follows: erlotinib (2 μM; LC Labs), 5-(N-ethyl-N-isopropyl) amiloride (EIPA) (10 μM; Sigma A3085), EGF (200 ng/mL; MCE HY-P7109). Antibodies against PRL3 (sc-130355), PRL1 (sc-130354), GST (sc-138) and c-myc (sc-40) were purchased from Santa Cruz Biotechnology. Antibodies against PI(3)P (Z-P003), PI(3,4)P_2_ (Z-P034B), PI(3,5)P_2_ (Z-P035) and PI(3,4,5)P_3_ (Z-P345B) were purchased from Echelon Biosciences. HRP-conjugated sheep anti-mouse (515-035-062) and goat anti-rabbit (111-035-045) antibodies were purchased from Jackson ImmunoResearch Laboratories Inc.

### Plasmids and Stable cell lines generation

Human embryonic kidney cell line HEK293T, Chinese hamster ovarian (CHO) cell line, human hepatoma cell line Huh7, human glioblastoma cell line U87 and U251 cells are ordered from ATCC. Chinese Hamster Ovarian (CHO) cells negative for all PRL isoforms were engineered to stably express Myc-Tagged PRL1 or PRL3 under the control of an inducible tetracycline (tet) promoter. The cell lines were established as previously described 62. PRL3 was knocked out using the CRISPR-Cas9 system as described previously 63. The sgRNA sequence (5'-GACCTATGACAAAACGCCGC-3') was ligated into the LentiCRISPR v2 vector via complementary oligonucleotides, and lentivirus particles were produced in HEK293T cells. Following lentiviral infection and puromycin selection, individual clones were selected and confirmed to be PRL3 knockout by Western blot analysis. Mixture pools of different clones were used for experiments. CHO cells were grown in RPMI 1640 medium supplemented with 10% serum, 1x L-Glutamine and 1% pen-strep antibiotic. U87, U251 and Huh7 cell lines were grown in DMEM supplemented with 10% serum, 1x L-Glutamine and 1% pen-strep antibiotic. For induction studies, 4µg/ml of doxycycline-HCl was added for 48 h. Cells with stable expression of pEGFP-C1 vector (Clontech), pEGFP-C1-PRL3 or pEGFP-C1-PRL1 were established as previous report 4.

### Western blot analysis

Cells were rinsed 2X with ice-cold PBS and lysed in RIPA buffer containing protease and phosphatase inhibitor cocktail (Pierce, Rockford, IL). Protein concentration was determined using a BCA assay (Pierce, Rockford, IL). Protein was denatured with Laemmli's buffer at 95 °C for 5 min and equal amounts of lysates were loaded to each well. Proteins were separated by SDS-PAGE gel electrophoresis and resolved proteins were transferred to PVDF membranes. Membranes were incubated in Tris-buffered saline containing 0.1% Tween-20 and 5% fat-free dry milk for 1 h at room temperature. Membranes were incubated with primary antibody for c-myc (9E10) (Santa Cruz Biotechnology, CA) overnight at 4 °C and subsequently with HRP-conjugated secondary antibody at room temperature for 1 h. Signals were visualized using ECL (Pierce, Rockford, IL) following the manufacturer's instructions.

### SEM sample processing

Cells plated on 12mm glass coverslips were allowed to grow till ~30-40% confluence. Tet-responsive protein expression was induced; cells were then fixed with 2% paraformaldehyde and 1% glutaraldehyde for 1.5 h. This step was followed by post-fixation with 1% osmium tetroxide for 1 h. The samples were then gradually dehydrated with a series of graded ethanol. The cells were then subjected to critical point drying using CO_2_ and subsequently sputter coated with gold. The samples were analyzed with a Philips XL-30 FEG scanning electron microscopy.

### Light microscopy

5x10^5^ cells seeded on 10 cm petri dish were allowed to grow to 40-50% confluence. The cells were then induced for the tet-responsive Myc-PRl1 and Myc-PRL3 protein expression. Images were then collected under light microscopy.

### Immunofluorescence

CHO cells plated on glass coverslips with 30-40% confluence were induced for myc-PRl1 and myc-PRL3 expression. Cells were then fixed in 3% paraformaldehyde for 20 min followed by permeabilization for 15 min in 0.1% saponin. Cells were incubated with c-Myc antibody for 1 h at room temperature followed by incubation with FITC-conjugated anti-mouse antibody (Molecular Probes) for 1 h. Following mounting, cells were viewed on a laser scanning head microscope.

### Immunohistochemistry (IHC)

CHO cells were fixed according to the procedures outlined in the Immunofluorescence section and were subsequently used for Immunohistochemistry (IHC) staining. Additionally, formalin-fixed and paraffin-embedded surgical specimens obtained from brain tumor patients were also prepared for IHC analysis. The specific steps of the immunohistochemistry protocol were described previously 64.

### Expression and purification of recombinant proteins in bacteria

cDNAs encoding PRL1, PRL3 or catalytically inactive mutant of PRL3, PRL3 CS (C104S) were cloned in frame with GST to the pGEXKG expression vector backbone to generate GST fusion forms of these proteins as described 65. The constructs were expressed in the *E. coli* strain DH5αF. The production of the fusion proteins was induced on incubation of the bacterial culture with 0.3 mM IPTG overnight at room temperature. The GST-fusion proteins were then extracted and eluted. Fractions were collected and analyzed by SDS-PAGE followed by coomassie staining for expression and purity.

### Lipid binding assay

The lipid binding assay was performed using PIP strips (Echelon Biosciences, Salt Lake City, UT) which are hydrophobic membranes spotted with 15 different lipids for interaction studies. The strips were incubated in blocking buffer (3 % fatty acid-free BSA in TTBS) overnight. This was followed by incubation with 1µg of purified GST, GST-PRL1, GST-PRL3 or GST-PRL3 CS fusion protein for 2 hrs at RT. The strips were then washed with TTBS and incubated with mouse anti-GST antibody (1:1000) for 1 h at RT. After several washes the membrane was then incubated with an anti-mouse HRP antibody (1:2000) for 1 h at RT followed by several washes with TTBS. The Signals were then visualized using the ECL.

### Phosphatase activity assay

Malachite green phosphatase activity assay (Echelon Biosciences, Salt Lake City, UT) was used to test for PRL protein phosphatase activity on different PIP substrates, the quantification of the released free phosphate product being the indicator of enzyme activity. The PIP substrates were reconstituted in water to a final concentration of 1 mM. The reaction was initiated in a 96-well plate by incubating 1 µg of each protein (GST, GST-PRL1 and GST-PRL3) separately with PIP substrates in reaction buffer (TBS with 10mM DTT) for 45 min at 37 °C. PTEN was used as a positive control (150ng). The samples were combined with 100 μL malachite green reagent and incubated for 20 min at RT followed by absorbance readings at 620 nm. The amount of inorganic phosphate released in each lipid sample was calculated based on a phosphate standard curve.

### PI(3)P ELISA

The quatification of PI(3)P Lipid changes was performed by using Echelon's PI3P Mass ELISA kit (K-3300) (Echelon Biosciences), according to the manufacturer's instructions.

### Docking studies

Molecular docking analysis was performed by AutoDock 4.2 66. Docking was processed with a 60 × 60 × 60 grid covering the region around the dimeric interface. The Lamarckian Genetic Algorithm (LGA) method was utilized and repeated for totally 10 runs. Other parameters were set as defaults.

### Dextran uptake assays

Cells were exposed to tetramethylrhodamine-conjugated dextran of 70 kD (TRM-DEX, Invitrogen D-1819), and subsequently, the fluorescence emitted by internalized dextran was evaluated using confocal microscopy. In parallel, for wild-type (WT) and PRL3 knockout (KO) cells, the fluorescence intensities of 10,000 cells per sample were quantified using a BD FACS cytometer (BD Biosciences).

### Glutamine deprivation assay

Cells were seeded in triplicate into 96-well plates (2 × 10^3^ cells/well) and cultured in complete medium for 24 h. Next, cells were washed once with PBS and incubated in medium containing 10% dialyzed FBS and 0.2 mM glutamine. Where indicated, 2% BSA was added. The medium was replaced every 24 h. After 6 d of culture, cell proliferation was measured using the ATPlite Luminescence Assay (PerkinElmer).

### Wound healing assays

5 x 10^4^ cells were seeded into μ-dishes (Ibidi, Martinsried) until they reached confluence. Inserts were discharged to create standardized 500 μm-wide gaps. The dishes were washed with PBS carefully and subsequently cultured in media with 0.5% FBS for 48 h. Images were observed under light microscope sequentially at every 24 hrs.

### Invasion assays

Briefly, 5 × 10^4^ cells (0.5 ml) were cultured in the upper chamber of the Matrigel Invasion Chambers with 8.0-μm pores (BD Biosciences). The lower chamber contained 0.75 ml media containing 1% serum. After 24 h, noninvasive cells in the upper chamber were removed by cotton swabs, and invasive cells were fixed and stained by the Hema 3 Stat Pack (Fisher Scientific) staining system. Cells were air-dried and peeled off to mount onto a slide with mounting buffer and then observed under a Leica inverted microscopy and then counted. Four different randomly chosen different fields in every triplicate of each sample were used for the statistical analysis.

### PRL3 gene expression and prognosis analysis

The publicly available gene expression and prognosis data were retrieved from The Cancer Genome Atlas (TCGA) and GTEx database. Survival curves and log-rank test statistics were analyzed via UCSC Xena web server (https://xenabrowser.net/) based on the TCGA datasets 67. The gene ontology analysis of PRL3 interactome proteins was performed by the String web server (www.string-db.org/), and visualized by Cytoscape software 68.

### Human studies

Human brain cancer samples were obtained with patient consent from the National University Hospital-National University of Singapore (NUH-NUS) Tissue Repository. Experimental procedures were approved by the Institutional Review Board (IRB) of NUH-NUS for research use and conducted in accordance with approved guidelines and regulations.

### Quantification and statistical analysis

The quantitative data of this study were presented as Means ± SD and analyzed using Student's t-test. (∗∗∗∗) P < 0.0001, (∗∗∗) P < 0.001 and (∗∗) P < 0.01. All data presented in this study are representatives of three biological independent experiments.

## Supplementary Material

Supplementary figure.
