# Supplementary material for: PRL1 and PRL3 promote macropinocytosis via its lipid phosphatase activity
Source: Theranostics. 2024 May 27;14(9):3423–38. doi: 10.7150/thno.93127 (PMC11209707; doi:10.7150/thno.93127)
Supplement: Supplementary file 1 — Supplementary figure. [file thnov14p3423s1.pdf]

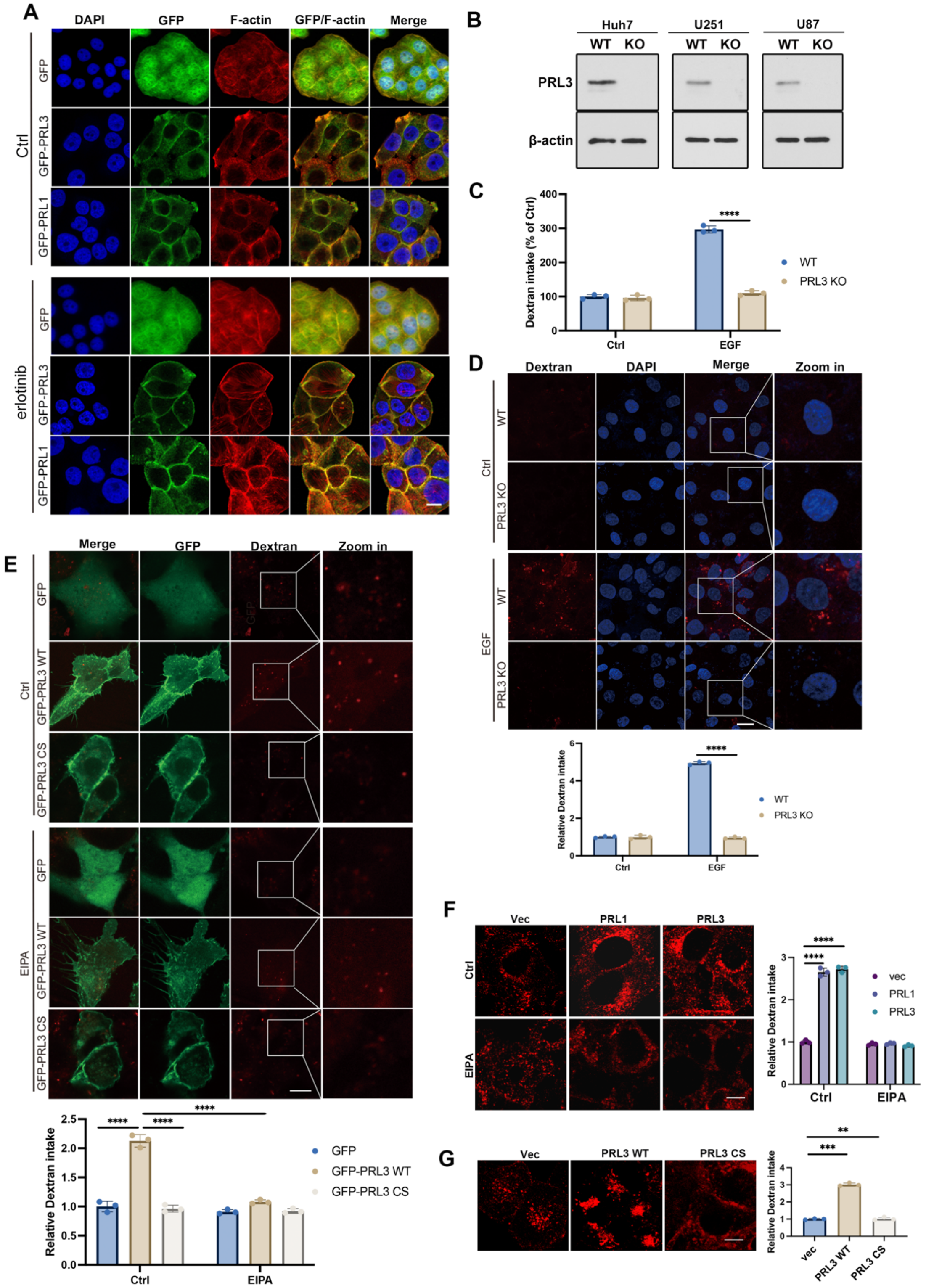

Figure S1. PRLs induce macropinocytosis depending on the phosphatase activity. (**A**) GFP, GFP-PRL1 and GFP-PRL3 overexpressing cells were treated with or without EGFR inhibitor erlotinib (2 μM). GFP and the IF signal of F-actin were observed under confocal microscope. Scale Bar, 10 μm. (**B**) The PRL3 expression were knockout in the Huh7, U87 and U251 cells. Cell lysate was subject to western blot analysis, which was performed with PRL3 and actin antibodies. (**C**) Dextran 70KD uptake assay was performed on WT and PRL3 KO U251 cells with or without treatment of EGF (200 ng/mL). Fluorescence intensity of 10,000 cells per sample was

determined by flow cytometry using the BD FACS cytometer. Three independent experiments were analyzed, means ±SD were presented. (**D**) WT and PRL3 KO Huh7 cells were treated as described in panel C and incubated with Dextran 70KD. Then cells were observed under confocal microscope. (**E-F**) Dextran 70KD uptake assay was performed on GFP, GFP-PRL3 WT and GFP-PRL3 CS mutant overexpressing U87 cells (panel E), and Vec, myc-PRL1, myc-PRL3 CHO cells (panel F). Cells were treated with or without macropinocytosis inhibitor EIPA. (**G**) Dextran 70KD uptake assay was performed on Vec, PRL3 WT and PRL3 CS mutant overexpressing CHO cells. Panel A, and D-G in this figure were examined by confocal microscopy (×600). Scale bar: 10 μm. The relative Dextran intake was analyzed by ImageJ, means±SD were presented.
